# Emerging Roles of the Tumor Suppressor p53 in Metabolism

**DOI:** 10.3389/fcell.2021.762742

**Published:** 2022-01-18

**Authors:** Lili Yu, Meng Wu, Gaoyang Zhu, Yang Xu

**Affiliations:** ^1^ Key Laboratory of Cancer Prevention and Intervention, Department of Medical Oncology, Ministry of Education, The Second Affiliated Hospital, Zhejiang University School of Medicine, Hangzhou, China; ^2^ Cancer Center, Zhejiang University, Hangzhou, China; ^3^ Cardiovascular Key Lab of Zhejiang Province, Department of Cardiology, The Second Affiliated Hospital, Zhejiang University School of Medicine, Zhejiang University, Hangzhou, China; ^4^ Shunde Hospital, Southern Medical University (The First People’s Hospital of Shunde), Foshan, China; ^5^ Division of Biological Sciences, University of California, San Diego, La Jolla, CA, United States

**Keywords:** p53, glucose metabolism, lipid metabolism, ferroptosis, amino acid metabolism, iron metabolism

## Abstract

Metabolism plays critical roles in maintaining the homeostasis of cells. Metabolic abnormalities are often considered as one of the main driving forces for cancer progression, providing energy and substrates of biosynthesis to support neoplastic proliferation effectively. The tumor suppressor p53 is well known for its roles in inducing cell cycle arrest, apoptosis, senescence and ferroptosis. Recently, emerging evidence has shown that p53 is also actively involved in the reprogramming of cellular metabolism. In this review, we focus on recent advances in our understanding of the interplay between p53 and metabolism of glucose, fatty acid as well as amino acid, and discuss how the deregulation of p53 in these processes could lead to cancer.

## Introduction

p53, encoded by the *TP53* gene, is a critical tumor suppressor that is required to prevent the oncogenic transformation of cells. Of note, *TP53* is the most frequently mutated gene in human cancers, and in most cases, *TP53* mutation is associated with poor prognosis (Levine, 2020; Olivier et al., 2010). Mutant p53 (Mutp53) proteins not only lose tumor suppressive functions, but also frequently acquire various gain-of-functions (GOF) that promote tumorigenesis. Under normal conditions, p53 is maintained in an inactive and unstable form through the interaction between p53 and its E3 ligase MDM2 and negative regulator MDMX (Fu et al., 2020). Under various stress conditions, p53 is stabilized and activated by post-translational modifications such as phosphorylation, acetylation, sumoylation, disrupting the interaction between p53 and Mdm2 and Mdmx (Fu, et al., 2020).

As a transcriptional factor, p53 directly activates and suppresses the transcription of hundreds of genes, many of which play key roles in cell cycle, apoptosis, and senescence (Vousden and Prives, 2009). For a long time, the roles of p53 in cell cycle arrest, apoptosis and senescence have been considered the major mechanisms to mediate its tumor suppressive activities (Vousden and Prives, 2009). However, the disruption of p53-dependent cell cycle arrest, apoptosis and senescence is not sufficient to induce cancer (Fu et al., 2020). Instead, various studies in mouse models such as the p53 (3KR/3KR) knock-in mouse model have highlighted its metabolic roles in inhibiting cancer progression (Li et al., 2012).

Reprogramming of cellular metabolism is one of the “hallmarks of cancer”, and is considered one of the main driving forces for tumorigenesis (Hanahan and Weinberg, 2011). In order to effectively support neoplastic proliferation, cancer cells increase their uptake of nutrients, especially glucose and amino acids, and adapt themselves to ensure their maximum utilization of the metabolic intermediates of glycolysis and oxidative phosphorylation for biosynthesis and NADPH production (Pavlova and Thompson, 2016). Numerous reports indicate that p53 is playing extensive and complex roles in regulating various metabolic pathways, and the gain of function mutants of p53 promotes the oncogenic metabolic reprogramming that induces drug resistance and metastasis.

In this review, we focus on recent advances in the research of p53 and its GOF mutants in regulating oncogenic metabolic alterations, aiming to provide insights into the targeted therapy of human cancers with metabolic regulation regiments.

## p53 and Glucose Metabolism

Numerous studies have shown that p53 plays complex roles in regulating glucose metabolism. Unlike normal cells, tumor cells use glucose mainly through glycolysis rather than oxidative phosphorylation (OXPHOS) to meet their energy and biosynthetic demand even under aerobic conditions, which is known as “Warburg effect” (Warburg et al., 1927). In many cases, p53 performs the tumor suppressive functions to inhibit aerobic glycolysis and promote OXPHOS.

p53 represses the transcription of glucose transporters GLUT1, GLUT3, and GLUT4 to reduce glucose uptake, which is the first rate-limiting event in glycolysis (Kawauchi et al., 2008; Schwartzenberg-Bar-Yoseph et al., 2004). p53 also transcriptionally induces TP53 Induced Glycolysis Regulatory Phosphatase (TIGAR) and inhibits 6-phosphofructo-2-kinase/fructose-2,6-bisphosphatase (PFKFB3 and PFKFB4), resulting in reduced intracellular levels of fructose-2,6-bisphosphate (F-2,6-BP), which functions as allosteric activator of phosphofructokinase (PFK), the rate-limiting enzyme catalyzing the conversion from F6P to F-1,6-BP (Bensaad et al., 2006; Franklin et al., 2016; Liu et al., 2020; Ros et al., 2017). Moreover, p53 was also reported to inhibit other glycolytic enzymes such as hexokinase 2 (HK2) and phosphoglycerate mutase 1 (PGAM1) (Kondoh et al., 2005; Wang et al., 2014). These findings support the notion that wild-type p53 suppresses glycolysis.

To further tilt the balance from glycolysis to OXPHOS, p53 also promotes cellular OXPHOS by various complementary mechanisms. p53 is able to inhibit the expression of pyruvate dehydrogenase kinase 2 (PDK2), a negative regulator of pyruvate dehydrogenase (PDH) that converts pyruvate to acetyl-CoA, leading to increased OXPHOS (Contractor and Harris, 2012). In addition, p53 induces the expression of Synthesis of Cytochrome C Oxidase 2 (SCO2), thereby promoting the synthesis of cytochrome C oxidase complex that catalyzes the major step of OXPHOS (Matoba et al., 2006). It was also reported that the induction of ferredoxin reductase (FDXR) by p53 promotes electron transfer from NADPH to cytochrome p450 (Liu and Chen, 2002). Moreover, p53 could promote mitochondrial biogenesis, support mitochondrial fission, maintain mitochondrial genome integrity, and ensure the quality control and turnover of mitochondria, thereby guarantees the proper function of mitochondria (Lacroix et al., 2020). Besides, the pentose phosphate pathway (PPP) is also reported to be repressed by p53 through its direct binding to glucose-6-phosphate dehydrogenase (G6PD), which is the first and rate-limiting enzyme of PPP. Consequently, p53 suppresses the production of NADPH as well as precursors for nucleotide biosynthesis (Jiang et al., 2011).

In contrast to the above reviewed canonical functions, the complexity of the roles of p53 in glucose metabolism remains to be elucidated. In this context, p53 could play an oncogenic role by dominantly suppressing OXPHOS. For example, in contrast to many types of human cancers such as lung cancer, wide-type p53 is often retained in hepatocellular carcinomas (HCC), where it induces PUMA expression to disrupt the oligomerization and function of mitochondrial pyruvate carrier (MPC) through direct PUMA-MPC interaction, thereby inhibits the mitochondrial pyruvate uptake and promotes glycolysis of HCC (Kim et al., 2019). These findings underscore the complexity of wild-type p53, indicating that the impact of p53 on glucose metabolism in cancer cells is complex and cell context dependent.

## p53, Lipid Metabolism and Ferroptosis

It has become increasingly clear that cancer cells gain the unique ability to synthesize fatty acids essential for cellular growth and survival (Beloribi-Djefaflia et al., 2016). Another non-canonical function of p53 is the capability to regulate lipid metabolism. p53 is thought to promote catabolism of fatty acids while simultaneously inhibit fatty acid synthesis. In addition to its inhibition of Glucose-6-phosphate dehydrogenase (G6PD) and pentose phosphate pathway (PPP) that is important for DNA synthesis and lipid synthesis (Jiang, et al., 2011), p53 can transcriptionally upregulate aromatase that is involved in lipid metabolism (Wang et al., 2013). Increased lipid accumulation in the livers of *p53* ^*−/−*^ mice is mitigated by the transgenic expression of aromatase, indicating important roles of p53-aromatase pathway in lipid metabolism (Wang, et al., 2013).

While wild-type p53 can suppress lipid synthesis by regulating the activities or levels of downstream effectors/targets such as G6PD and aromatase (Jiang, et al., 2011; Wang, et al., 2013), numerous reports have demonstrated that mutant p53 can promote lipid synthesis by altering the activities of various transcription factors or signaling molecules such as p63, p73, Nrf2, and AMP-activated protein kinase (AMPK), which are involved in lipid metabolism (Do et al., 2012; Walerych et al., 2016; Xu et al., 2011). Several studies have shown that the upregulation of enzymes involved in the synthesis of fatty acids and cholesterol (mevalonate pathway) is required for tumor progression (Bathaie et al., 2017; Kuhajda et al., 1994; Ribas et al., 2016; Roongta et al., 2011; Zhan et al., 2008). The presence of p53 mutations correlates with high levels of enzymes involved in the mevalonate pathway in human breast cancer tissues (Freed-Pastor et al., 2012). Another study shows that ectopic expression of p53 mutants (p53^R175H^ and p53^P151S^) inhibits AMPK activity and subsequently reduces phosphorylation of Acetyl-CoA carboxylase (ACC) under glucose and serum starvation in a p53-null head and neck squamous cell carcinoma (HNSCC) cell line UMSCC1 (Zhou et al., 2014).

Ferroptosis is a new form of programmed cell death characterized by the accumulation of iron-dependent lethal lipid peroxides (Dixon et al., 2012). p53 plays an important role in modulating ferroptotic responses by regulating the expression of its metabolic targets (Jiang et al., 2015). For example, recent studies have shown that ALOX12 is critical for p53-mediated ferroptosis (Chu et al., 2019). In addition, p53 induces ferroptosis partly through transcriptional activation of Glutaminase 2 (Jennis et al., 2016) and SAT1 (a polyamine catabolic enzyme) (Ou et al., 2016), and transcriptionally represses *SLC7A11* (Jiang, et al., 2015). In addition, suppressor of cytokine signal transduction protein 1 (SOCS1) is required for p53-mediated expression of p53 target genes involved in ferroptosis. In this context, SOCS1 can reduce the expression of SLC7A11 to sensitize cells to ferroptosis (Saint-Germain et al., 2017). However, p53 behaves differently in a context dependent manner. While the basal p53 promotes ferroptosis, stress-induced p53 can inhibit ferroptosis (Tarangelo et al., 2018; Xie et al., 2017). For example, p53 inhibits ferroptosis by inhibiting dipeptidyl-peptidase-4 (DPP4) activity in human colorectal cancer cell lines (Xie, et al., 2017). Therefore, further studies would be needed to clarify the complex roles of p53 in ferroptosis.

## p53 and Iron Metabolism

In addition to ferroptosis, p53 also modulates iron homeostasis. p53 expression is decreased upon the exposure to excessive levels of iron through heme-p53 interaction (Shen et al., 2014). Under iron-deprived conditions, HIF1α is activated to increase p53 protein stability and protein levels (An et al., 1998; Peyssonnaux et al., 2008; Peyssonnaux et al., 2007). In contrast, p53 is also found to be downregulated upon iron depletion via MDM2 (Dongiovanni et al., 2010). Therefore, the regulatory mechanisms of p53 by the iron concentration appear to be context dependent.

p53 can control the intracellular iron pool by modulating the expression of iron sensors. For example, p53 directly activates the expression of hepcidin, an iron-regulating hormone (Weizer-Stern et al., 2007). Another study suggests that p53 induces the expression of iron-sulfur cluster assembly proteins (ISCU) and protects cells from iron overload (Funauchi et al., 2015). p53 has been reported to modulate mitochondrial proteins that are involved in iron metabolism. For example, p53 mediates the expression of its target ferredoxin reductase (FDXR), and subsequently, modulates mitochondrial iron homeostasis through iron sulfur clusters (ISC) or heme synthesis (Sheftel et al., 2010).

## p53 and Amino Acid Metabolism

Amino acid metabolism has extensive effects on tumors, and it has been revealed that p53 functions to protect cells from metabolic stress and promote cellular survival. Cancer cells rely on glutamine for cellular proliferation after glucose depletion through a process named glutaminolysis, by which glutamine is converted to the intermediates of the TCA cycle (Pavlova and Thompson, 2016). p53 activates the expression of Glutaminase 2 (GLS2), a key enzyme in glutamine-based cellular energy production under glucose-deprivation conditions to support cancer cell growth. Glutamate also limits intracellular and extracellular oxidative stress to promote cell survival (Suzuki et al., 2010).

Under the conditions when both glucose and glutamine become limited, aspartate metabolism becomes very important for cellular energy production. p53 is reported to transactivate Solute Carrier Family 1 Member 3 (SLC1A3), an aspartate/glutamate transporter, under glutamine starvation conditions (Tajan et al., 2018). p53 can also promote cellular survival by the induction of high affinity amino acid transporter Solute Carrier Family 1 Member 3 (SLC1A3) (Tajan, et al., 2018). Another important player in tumor cell survival and proliferation is serine. p53 promotes serine synthesis by glutathionine (GSH) synthesis, eventually leading to overall cell survival (Maddocks et al., 2013). In summary, p53 promotes cellular survival by promoting energy production from amino acids under the condition of glucose deprivation.

## Concluding Remarks

**FIGURE 1:**
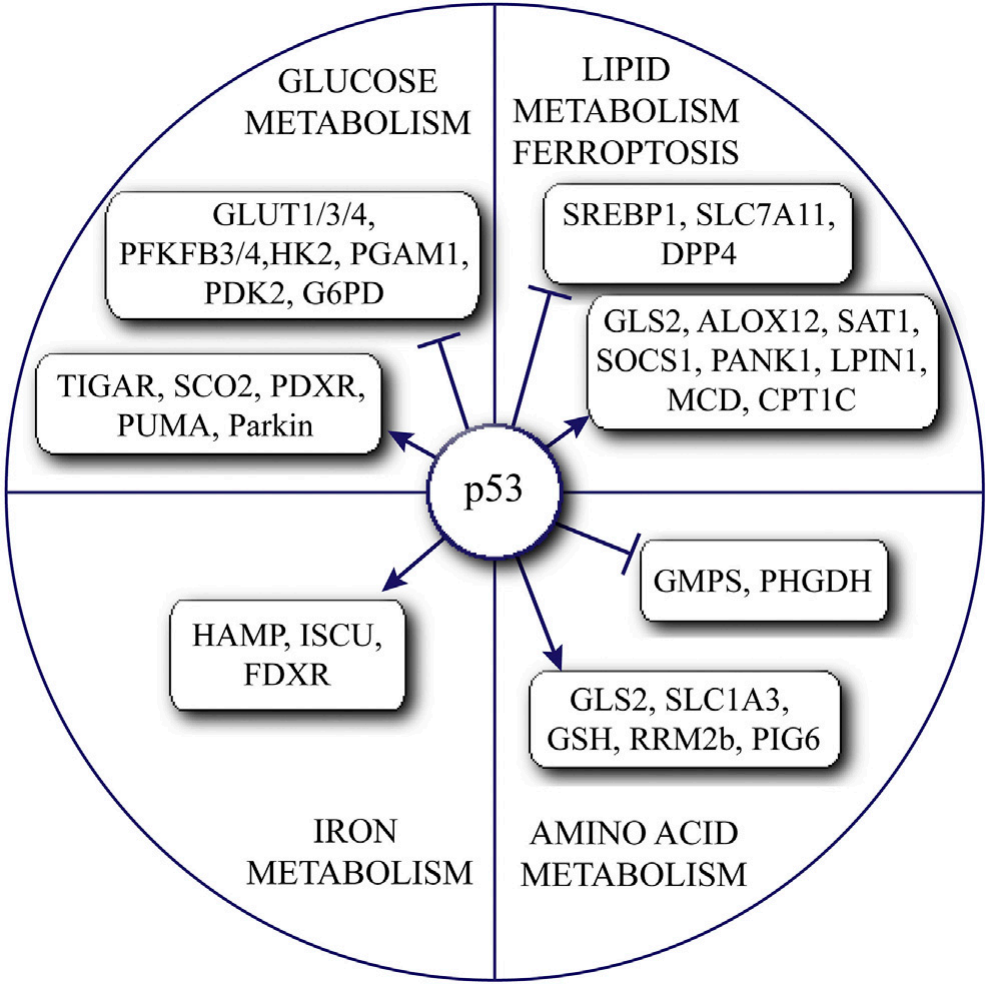
Summary of the complex roles of p53 in regulating various metabolic pathways.

Tumor suppressor gene p53 is not only essential in cell cycle arrest or apoptosis, but also participates in various physiological functions. Here we take a closer look at the complexity of p53 function in regulating cellular metabolism. These findings together suggest that p53 could regulate various aspects of cellular metabolism via regulating different target gene expression or protein-protein interactions in a cellular and environmental context dependent manner (Figure 1). The roles of wild-type p53 in tumor metabolism are complex, and sometimes, could conflict with its status as a tumor suppressor. For example, some roles of p53 in suppressing OXPHOS and inducing amino acid based energy production can promote cancer cell survival and proliferation (Kim, et al., 2019; Suzuki, et al., 2010, Tajan et al., 201). While the full-length p53 mutants are found to be overexpressed in more than half of human cancers and apparently gain new oncogenic properties (Zhu et al., 2020), many questions remain unanswered for their roles in cellular metabolism. Further advancements in single-cell analysis and multi-omics analyses will provide more in-depth understanding of p53-related regulatory mechanisms.

Considering the unusual reliance of cancer cells on glycolysis, targeting tumor metabolic reprogramming has become a promising strategy for cancer treatment. In addition, the increased glycolysis contributes to higher levels of the acidic intermediates such as lactate and acidic tumor microenviroment, directly or indirectly suppress tumor immunity. Therefore, the activation of the roles of p53 in suppressing the metabolic reprogramming of cancer cells could become effective targeted therapy for human cancers. However, the development of such strategy requires attention to the complex and sometimes conflicting roles of p53 in cancer cells. The comprehensive understanding of various p53 regulated pathways will enable the precise activation of the p53-dependent pathways in suppressing tumor metabolism.
